# The relationship between smoking and rosacea: A Mendelian randomization study

**DOI:** 10.1111/jocd.16498

**Published:** 2024-08-13

**Authors:** YuJia Cai, HaiFeng Zeng, MaoCan Tao

**Affiliations:** ^1^ Department of Cosmetic Dermatology The First Affiliated Hospital of Zhejiang Chinese Medical University Hangzhou China; ^2^ Zhejiang Provincial Hospital of Chinese Medicine Hangzhou China

**Keywords:** Mendelian, rosacea, smoking

## Abstract

**Background:**

Rosacea can be seen in many patients nowadays, and the related causes are complex. Despite a certain association between smoking and rosacea being reported by several studies, the actual causality has not been established for the possible bias and confounders.

**Methods:**

We used Mendelian randomization (MR) to evaluate a potential causal effect of smoking on rosacea risk. Statistics on smoking and rosacea were obtained from the FinnGen project and Neale Lab Consortium. The causal association was assessed by multiple methods including inverse variance weighted (IVW), MR Egger, weighted median, and weighted mode. Furthermore, sensitivity analyses were also conducted to address pleiotropy, along with the leave‐one‐out method.R version 4.2.3 was applied for the analyses.

**Results:**

The IVW estimation revealed that previous smoking has a deleterious effect on rosacea (odds ratio [OR] = 6.7729, 95% confidence interval [CI] = 1.5691–29.2356, *p* = 0.0104). By contrast, there was no statistically relationship between current smokers and rosacea (OR = 0.6180, 95% CI = 0.0605–6.3094, *p* = 0.6847). Results were similar in the analysis based on the weighted median method (previous smoking: OR = 8.6297, 95% CI = 1.0131–73.5071, *p* = 0.0486; current smoking: OR = 0.2896, 95% CI = 0.0106–7.9132, *p* = 0.4627). The stability of the causal effect estimates was supported by several sensitivity analyses and the leave‐one‐out method.

**Conclusion:**

Our MR study found support forrosacea risk and previous smoking. Although no evidence was found to increase the risk of rosacea in current smokers, to prevent various diseases associated with smoking, the public should be encouraged to avoid smoking at the very beginning.

## 1 INTRODUCTION

Rosacea is regarded as a chronic inflammatory disease predominantly affecting the centrofacial region and the eyes. It usually starts at the age of 30–50, but could happen at any time in fact.^1^, ^2^ The common characteristics of rosacea include flushing, persistent erythema, telangiectasia, papules/pustules, and phymatos. Phymatos changes are mostly occurring at the nose, which is also known as rhinophyma, and are more frequently seen in men.^3^ According to its clinical manifestation, rosacea could be divided in to three types: erythematotelangiectatic rosacea (ETR), papulopustular rosacea (PPR), and phymatous rosacea (PhR), and ETR could be the most common one.^4^, ^5^ As a discosmetic dermatosis, different types of rosacea could impact the quality of life and mental health in different degrees, thus lead to anxiety, depression, and self‐abasement.^6^, ^7^, ^8^ Therefore, it is of great importance to avoid the occurrence of rosacea at its source. The causes of rosacea are complex, as an inflammatory skin disease, external environmental stimuli such as sun exposure and high temperature, internal environmental transformation of the human body such as immune diseases and mental disorders, and daily lifestyle including eating habits, smoking, drinking, and sleeping habit, could all disrupt the homeostasis of skin and lead to the development of rosacea.^9^, ^10^

It is worth noting that several epidemiological studies have explored the association between smoking and rosacea, but some reported conclusions seemed to be controversial. In addition, two studies of meta‐analyses both showed an interesting conclusion, indicating that smoking status, current smokers and ex‐smokers, had diametrically opposite effects on the risk of developing rosacea.^11^, ^12^ However, almost all the data used in the two meta‐analyses on smoking in relation to rosacea came from observational studies, which cannot completely rule out distortions. Therefore, the reliability of these findings still remains skeptical.

**FIGURE 1:**
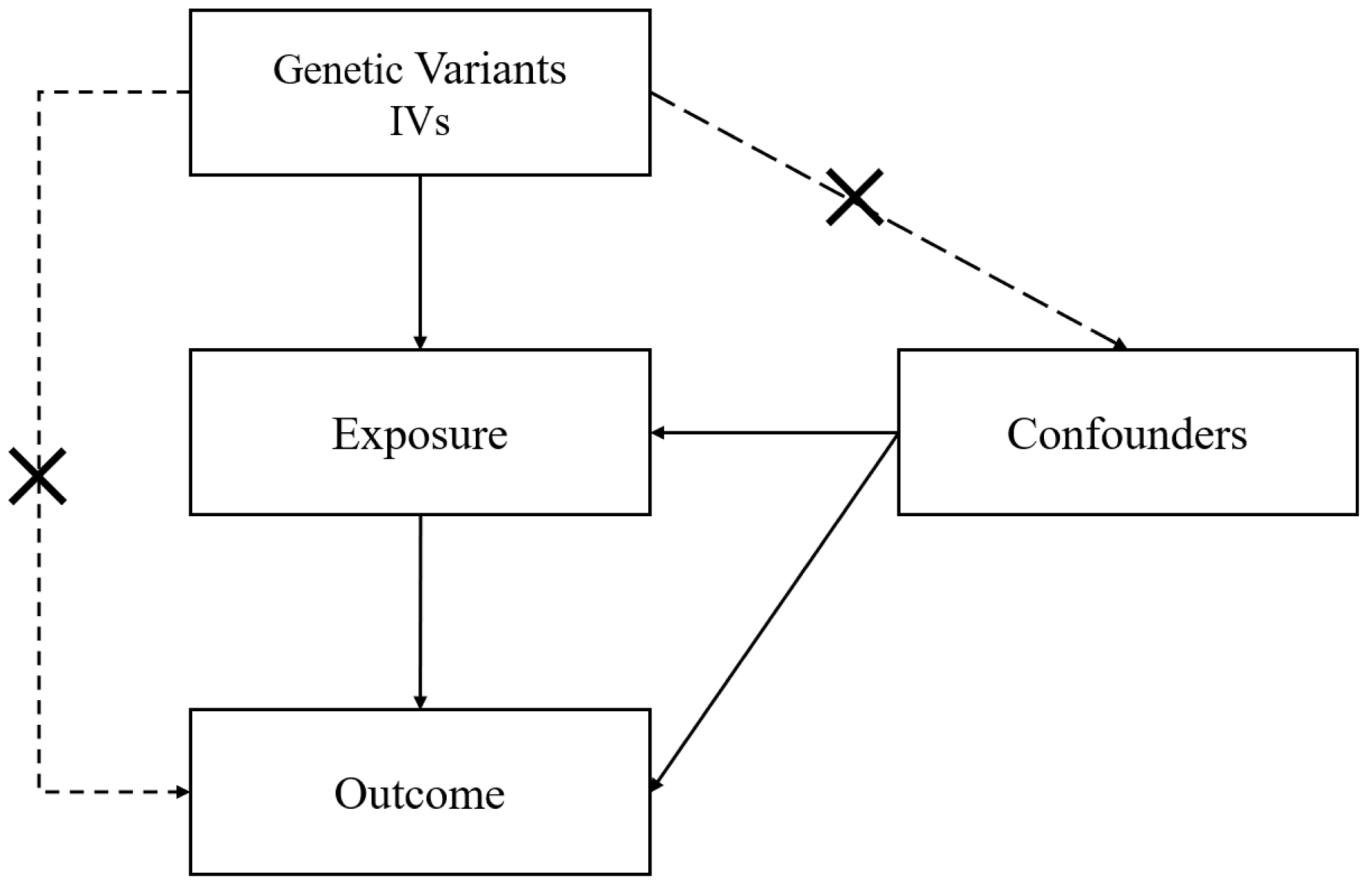
Conceptual framework diagram for the MR analysis.

Single‐nucleotide polymorphisms (SNPs) are an example of a genetic variant that can be used as an instrument variable (IV) in Mendelian randomization (MR), an efficient and effective technique that strengthens the causal inference in the association between exposure and outcome.^13^, ^14^ Since genetic variants are randomly distributed during conception, MR is not susceptible to confounding and is therefore unrelated to certain confounders, including behavioral and environmental factors.^15^ Three conditions must be satisfied for MR to be deemed robust: (1) the genetic variations must be linked to the exposure, (2) the variants must not be linked to any other confounders, and (3) the variants must only be linked to the outcome through the relevant exposure (Figure 1).^16^

Thus, we performed a multivariate regression analysis (MR) using genome‐wide association studies (GWAS) data derived from two populations in order to elucidate the precise relationship between smoking and rosacea, particularly with regard to varying smoking status, and to enable improved causative management.

Therefore, to clarify the exact relationship between smoking and rosacea, especially different smoking status, and for applying better causal management, we conducted an MR analysis on genome‐wide association studies (GWAS) data obtained from two samples.

## 2 MATERIALS AND METHODS

### 2.1 Data sources

GWAS data for rosacea (ICD9 code 6953A, ICD10 code L71.8, L71.9) were retrieved from the FinnGen database (http://www.finngen.fi), including 1195 cases and 211 139 controls, and the corresponding phenotype code was “L12_ROSACEA”. Meanwhile, GWAS data for two smoking statuses, “smoking status: previous” (118 419 cases and 217 605 controls) and “smoking status: current” (33 928 cases and 302 096 controls) were obtained from the Neale Lab Consortium, and the used dataset were “ukb‐a‐224” and “ukb‐a‐225”. Data about the two types of smoking status were from the same sample with 16 380 452 SNPs and all the participants above were European.

In addition, ethical approval or patient permission was not necessary because all of the data used in this investigation came from publically accessible sources.

### 2.2 Instrumental variables selection

To identify IVs, the subsequent standards were utilized: First, SNPs associated with the exposure were identified if *p* < 5 × 10^−6^; second, linkage disequilibrium (LD) interference was excluded by setting a *r* ^2^ threshold of 0.001 and a clump distance of 10 000 kb; third, SNPs with minor allele frequency (MAF) ≤0.01 were eliminated; and fourth, palindromic SNPs were eliminated to prevent bias.

Finally, a total of 94 SNPs associated with “smoking status: previous” and 93 SNPs related with “smoking status: current” at the genome‐wide significance threshold were identified by the GWAS. After the removing of SNPs for being unavailable or being palindromic with intermediate allele frequencies (rs1492546, rs4971088, rs7596680, rs10741743), there were 88 independent SNPs left as IVs for “smoking status: previous” and 91 for “smoking status: current” in the end.

### 2.3 Statistical analysis

The primary statistical analysis approach we employed was the inverse‐variance weighted (IVW) method; however, in order to acquire more thorough and precise results, we also ran MR‐Egger, weighted median (WM), and weighted mode analyses. The most widely used MR approach, the IVW method, assumes the validity of all instruments and distributes weights based on the inverse variance of each IV.^17^ The slope can be understood as the smoking behavior influence on rosacea modified for horizontal pleiotropy. The MR‐Egger technique uses a type of weighted linear regression analysis, which is more robust against erroneous IVs.^18^ The MR‐PRESSO test can also be used to find out if pleiotropy is present, locate outlier SNPs, and rerun studies to provide results that are exactly the same as those from IVW once outliers have been eliminated. The weighted median estimator may yield a meaningful causal estimate when at least half of the instruments are valid. Similarly, the weighted mode analysis estimate is legitimate if the biggest collection of instruments with consistent MR estimates is effective.^19^ Heterogeneity was also evaluated; the absence of heterogeneity is indicated by a *p* value >0.05. Additionally, sensitivity analysis was carried out with the leave‐one‐out technique. Utilizing the 95% confidence interval (CI) and OR, the degree of effect was assessed.

The analyses were performed with R version 4.2.3, utilizing the “TwoSampleMR”^20^ and “MRPRESSO”^18^ packages.

## 3 RESULTS

**FIGURE 2:**
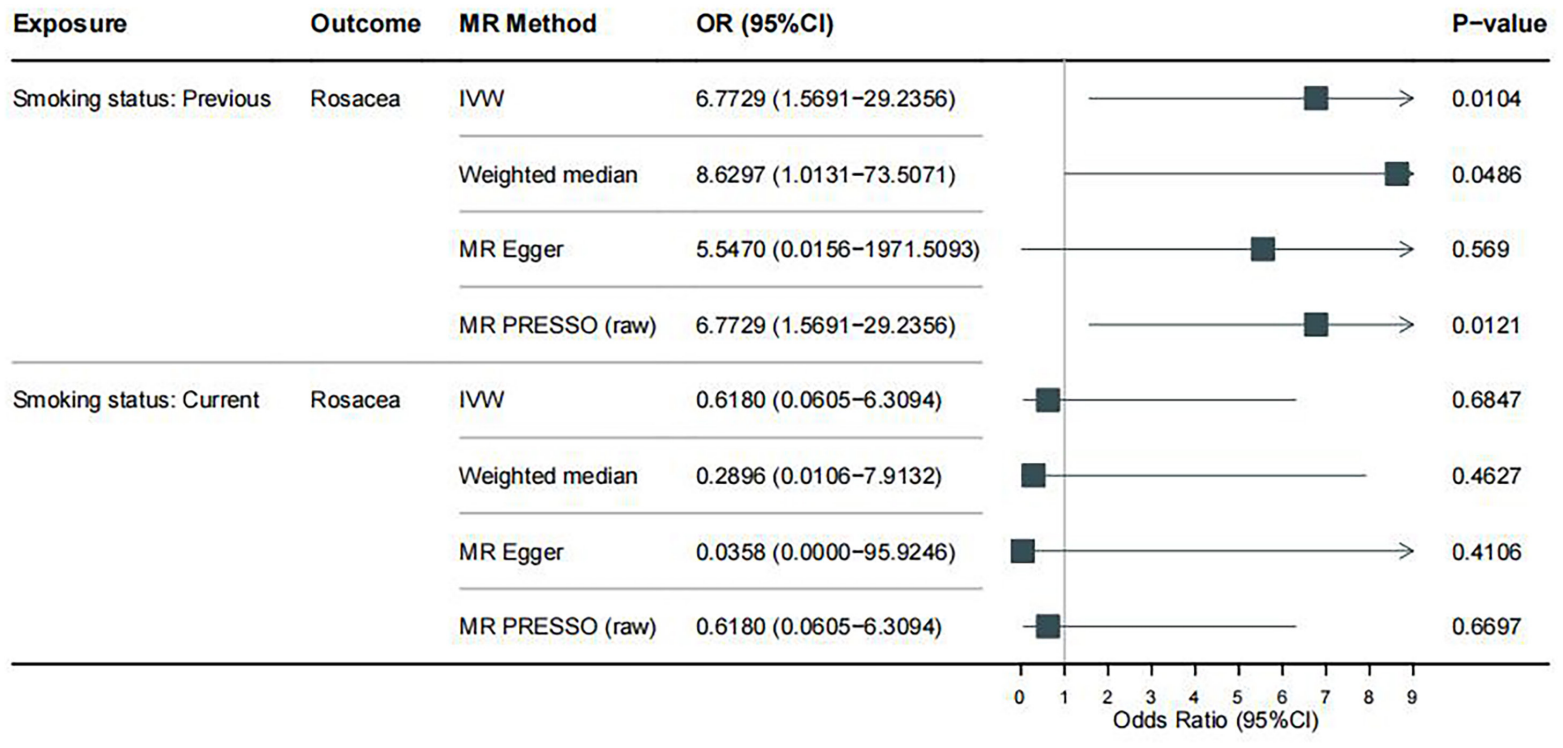
Forest plot of MR results. IVW, inverse‐variance weighted; MR, Mendelian randomization; OR, odds ratio; PRESSO, pleiotropy residual sum and outlier.

**FIGURE 3:**
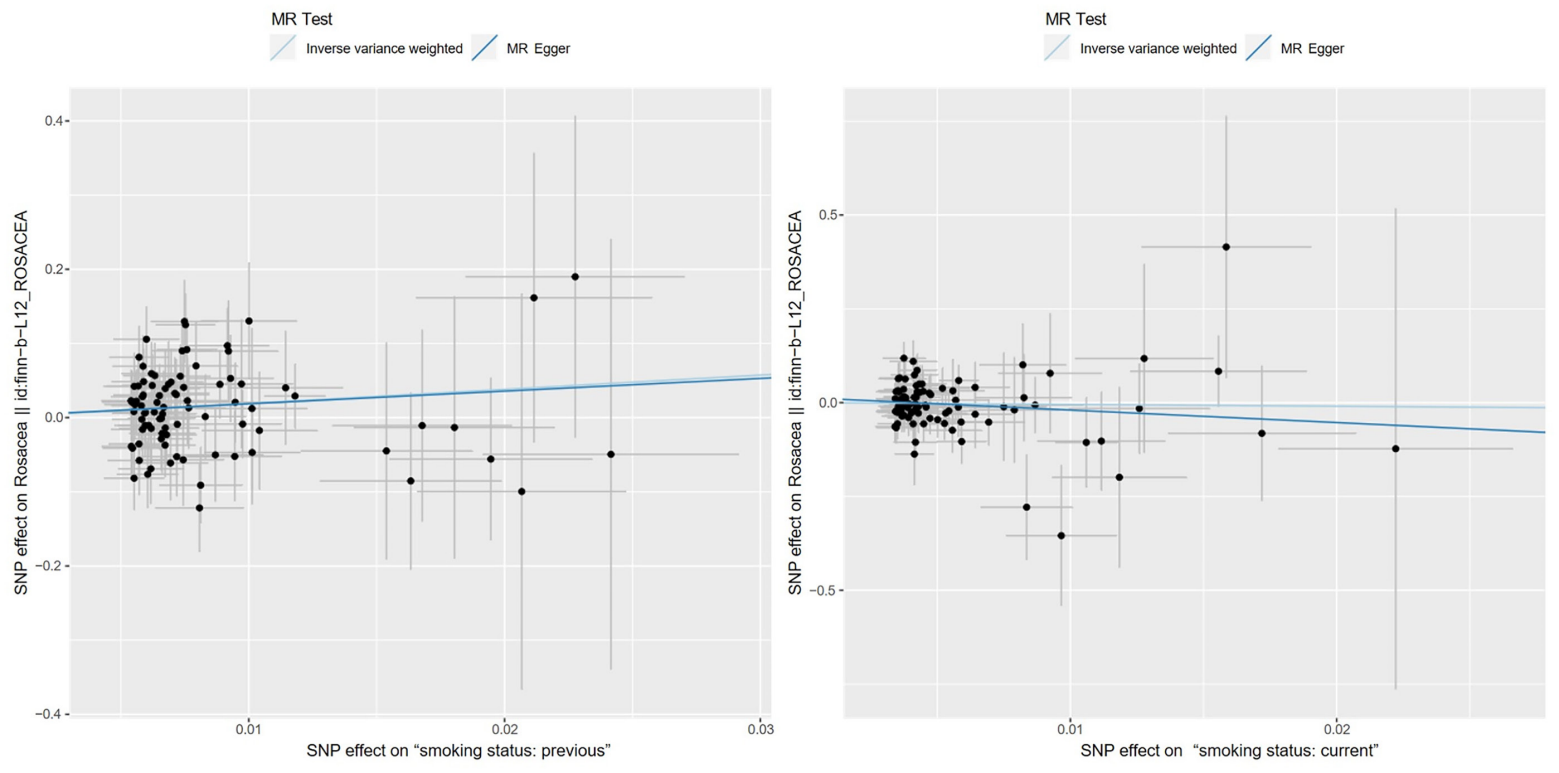
Scatter plot of the causal relationship between smoking and rosacea.

The main results of our study were shown in a forest plot (Figure 2) and a scatter plot (Figure 3), indicating an increased risk of rosacea in ex‐smokers while not in current smokers. The IVW analysis revealed that previous smoking might have a deleterious effect on rosacea (OR = 6.7729, 95% CI = 1.5691–29.2356, *p* = 0.0104), however, there was no statistically relationship between current smokers and rosacea (OR = 0.6180, 95% CI = 0.0605–6.3094, *p* = 0.6847).Results were similar in the analysis based on the weighted median method: previous smoking: OR = 8.6297, 95% CI = 1.0131–73.5071, *p* = 0.0486; and current smoking: OR = 0.2896, 95% CI = 0.0106–7.9132, *p* = 0.4627.

**FIGURE 4:**
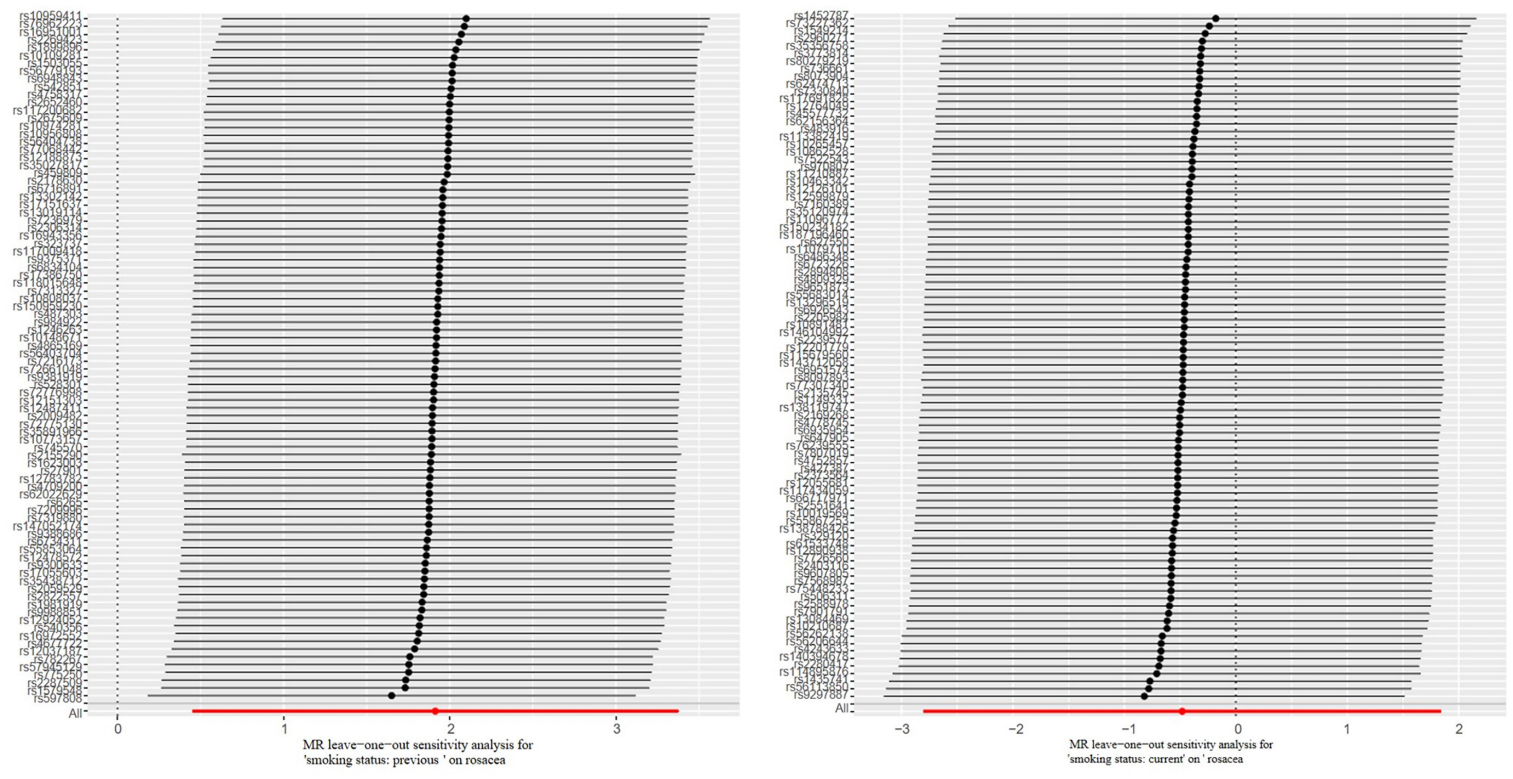
Leave‐one‐out plots for the causal association between smoking and rosacea.

In addition, heterogeneity and pleiotropy results are presented in Table 1. As for heterogeneity, no statistical significance was found both in previous smoking (*p* = 0.4254) and current smoking (*p* = 0.7293). Similarily, the results showed no statistical significance in MR Egger pleiotropytest (*p* = 0.9453 for previous smoking and*p* = 0.4612 for current smoking), indicating the reliability of the results. Besides, as no outlier was found in MR‐PRESSO global outlier test, the OR and 95% CI values of MR‐PRESSO test were the same as IVW analysis. Meanwhile, the stability of the causal effect estimates was also proved by the leave‐one‐out method, which was showed in Figure 4, have confirmed the stability and reliability of the causal effect estimates.

**TABLE 1:** Sensitivity analyses of MR analysis.

Exposure	Outcome	SNPs	Heterogeneity test		MR Egger pleiotropy test		MR‐PRESSO global outlier test
			*Q*	*p*‐Value	Intercept	*p*‐Value	Outlier
Smoking status: Previous	Rosacea	88	87.8143	0.4254	0.0015	0.9453	None
Smoking status: Current	Rosacea	91	80.4677	0.7293	0.0135	0.4612	None

Abbreviations: MR, Mendelian randomization analysis; SNPs, Number of single nucleotide polymorphism.

## 4 DISCUSSION

We used data from the FinnGen project and the Neale Lab collaboration collaboration to conduct a two‐sample MR analysis in an attempt to determine the causal link between smoking and rosacea. No statistically significant correlation was discovered between present smoking and rosacea, despite the study's genetic support for a causal relationship between prior smoking and the condition.

It is worth noting that both Zhang and Yuan^11^, ^12^ indicated that ex‐smokers showed an increased risk of rosacea in their studies, which were consistent with our findings. According to the widely accepted theory, cigarette smoking has been associated with significant morbidity affecting different systems of the body, as one of the main constituents of cigarette smoke, nicotine could cause microvascular vasoconstriction, which might benefit in decreasing the development of rosacea. In addition, nicotine could also suppresses the immune system, which may help reduce the inflammatory response.^21^ Therefore, the immune inflammatory response of individuals is suppressed when smoking, and a rebound vasodilatation and immune activity could occur after nicotine withdraw, these reactions could contribute to the development of rosacea, which might explain the increased risk of rosacea among ex‐smokers.^22^ Current smoking appears to be beneficial for reducing the risk of rosacea based on the mechanisms mentioned above, but our results showed there was no significance association between them. Zhang's^12^ study revealed that smoking was only associated with the occurrence of PhR and not ETR or PPR, despite some studies suggesting that smokers who are actively quitting may have a lower chance of developing rosacea.

The precise connection is still up for debate because these findings are based on observational research, which may be influenced by confounding factors. In addition to weakening the immune system, smoking has also been demonstrated to produce more pro‐inflammatory cytokines while lowering the amounts of anti‐inflammatory cytokines,^23^ which is in contradiction with the mechanism mentioned above, and it could lead to the increased risk of rosacea. On the other hand, cigarette smoke could also modify diversifying of keratinocytes as well as accelerate the degradation of elastic fibers and collagen, which could damage the basic skin structure and destroy the skin barrier potentially, promoting the development of rosacea.^24^ According to the above, the effect of smoking on the skin is actually a combination of various advantages and disadvantages. We believe that the reduced risk of rosacea among current smokers could be temporary, smoking does not have a positive effect on rosacea in the long run, which is similar to Yuan's viewpoints.^11^ In addition, various lifestyle factors might be correlated as the definition of smoking is intricate, making the test of horizontal pleiotropy especially important. No evidence of pleiotropic effects was found by conducting various sensitivity analyses, which made our results more reliable.

There are some strengths of this study. MR design is the major strength of our study, it could minimize bias and reverse causality, and used sensitivity analysis to ensure reliability of the results, improving the causal inference in the associations between smoking status and rosacea in our study. In addition, as the population of our analysis was confined within the European ancestry, the deviation of population stratification can be effectively minimized, making the results more stable and reliable. However, our study also has several limitations that we could not ignore. Since the study was confined to populations of the European and there may be some genetic differences between different races, it is unknown if the observed results can be applied to other populations, and it was also not possible to do the sex‐based stratification analysis. Another drawback is that without individual‐level data, we were unable to examine nonlinear correlations, and conducting subgroup analysis based on rosacea subtypes, daily cigarette consumption, and years since smoking start or stop was unlikely. Although sensitivity analyses proved the reliability and stability of our results, those residual pleiotropy mentioned above is difficult to exclude and should be considered if possible. Therefore, researches include high‐quality GWASs of diverse subgroups are still required.

In summary, this MR analysis used summary statistics and showed that ex‐smoking was associated with an increased risk of rosacea, and no statistical significance association between current smoking and rosacea was proved. The results doesn't mean that we should keep smoking to avoid developing rosacea, on the contrary, smoking does no good to human health in the long run as several Mendelian studies have found the connection between smoking exposure and various diseases, such as gastrointestinal diseases and stroke.^15^, ^25^ Consequently, it is essential to avoid smoking from the very beginning.

## 5 CONCLUSION

In conclusion, this MR study provides supportive evidence for a potential causal association between previous smoking and increased risk of rosacea, but not between current smoking and rosacea. To prevent diseases related to smoking, the public should be encouraged to avoid smoking.

## AUTHOR CONTRIBUTIONS

Yujia Cai drafted the article. HaiFeng Zengwas responsible for data curation and validation. MaoCan Tao gave the final approval of the version to be submitted. All authors read and approved the final version of the manuscript.

## CONFLICT OF INTEREST STATEMENT

The authors declare no conflict of interest.

## Data Availability

The data that support the findings of this study are available in [GWAS summary datasets] at [https://gwas.mrcieu.ac.uk/], reference number [L12_ROSACEA, ukb‐a‐224 and ukb‐a‐225]. These data were derived from the following resources available in the public domain: [FinnGen database and Neale Lab Consortium; http://www.finngen.fi].
